# Serum 1H-NMR Metabolomic Fingerprints of Acute-On-Chronic Liver Failure in Intensive Care Unit Patients with Alcoholic Cirrhosis

**DOI:** 10.1371/journal.pone.0089230

**Published:** 2014-02-19

**Authors:** Roland Amathieu, Mohamed N. Triba, Pierre Nahon, Nadia Bouchemal, Walid Kamoun, Hakim Haouache, Jean-Claude Trinchet, Philippe Savarin, Laurence Le Moyec, Gilles Dhonneur

**Affiliations:** 1 Service d’Anesthésie et des Réanimations Chirurgicales, Université Paris 12, Hôpital Henri Mondor, Assistance Publique des Hôpitaux de Paris (AP-HP), Créteil, France; 2 Universite Paris 13, Sorbonne Paris Cité, Laboratoire Chimie, Structures, Propriétés de Biomatériaux et d’Agents Thérapeutiques (CSPBAT), Unité Mixte de Recherche (UMR) 7244, Centre National de Recherche Scientifique (CNRS), Equipe Spectroscopie des Biomolécules et des Milieux Biologiques (SBMB), Bobigny, France; 3 Service d’Hépatologie et Université Paris 13, Hôpital Jean Verdier, Assistance Publique des Hôpitaux de Paris (AP-HP), Bondy, France; 4 Institut National de la Santé et de la Recherche Médicale (INSERM) Unité de Biologie Intégrative des Adaptations à l’Exercice (UBIAE U902), Université d’Evry, Evry, France; Mayo Clinic, United States of America

## Abstract

**Introduction:**

Acute-on-chronic liver failure is characterized by acute deterioration of liver function in patients with compensated or decompensated, but stable, cirrhosis. However, there is no accurate definition of acute-on-chronic liver failure and physicians often use this term to describe different clinical entities. Metabolomics investigates metabolic changes in biological systems and identifies the biomarkers or metabolic profiles. Our study assessed the metabolomic profile of serum using proton nuclear magnetic resonance (^1^H-NMR) spectroscopy to identify metabolic changes related to acute-on-chronic liver failure.

**Patients:**

Ninety-three patients with compensated or decompensated cirrhosis (CLF group) but stable liver function and 30 patients with cirrhosis and hospitalized for the management of an acute event who may be responsible of acute-on-chronic liver failure (ACLF group), were fully analyzed. Blood samples were drawn at admission, and sera were separated and stored at –80°C until ^1^H-NMR spectral analysis. Using orthogonal projection to latent-structure discriminant analyses, various metabolites contribute to the complete separation between these both groups.

**Results:**

The predictability of the model was 0.73 (Q^2^ *Y*) and the explained variance was 0.63 (R^2^ *Y*). The main metabolites that had increased signals related to acute-on-chronic liver failure were lactate, pyruvate, ketone bodies, glutamine, phenylalanine, tyrosine, and creatinine. High-density lipids were lower in the ALCF group than in CLF group.

**Conclusion:**

A serum metabolite fingerprint for acute-on-chronic liver failure, obtained with ^1^H-NMR, was identified. Metabolomic profiling may aid clinical evaluation of patients with cirrhosis admitted into intensive care units with acute-on-chronic liver failure, and provide new insights into the metabolic processes involved in acute impairment of hepatic function.

## Introduction

Acute-on-chronic liver failure (ACLF) is a syndrome characterized by acute deterioration of liver function in patients with compensated or decompensated, though stable, cirrhosis. ACLF is a serious condition that has varied etiologies and manifestations, as well as a high mortality rate [1]. ACLF commonly occurs after an acute event, and is often associated with extra-hepatic organ failure. The most common triggers for ACLF include sepsis and digestive bleeding [2]. Any patient with cirrhosis is exposed to acute complications, which, in turn, may occasionally initiate a cascade of events that can lead to further deterioration of liver function, multiple-organ failure, and death within a few days or weeks. These patients with cirrhosis need ICU management.

There is no consistent or clear definition of ACLF in the literature, and physicians often use this term to describe different conditions. Previous studies on ACLF have used different definitions, and there is no overall consensus for the liver failure that occurs, for the causative acute event, or for the diagnosis of the underlying chronic liver disease. Recently, the Asia-Pacific Association for the study of liver disease and the EASL-CLIF consortium have proposed different consensus definition for ACLF [3], [4]. In the Asiatic consensus, criteria for defining ACLF vary widely from the level of bilirubin, to the time until deterioration after onset, to an initiating event that is accepted as jaundice, or to any other symptom that pertains to hepatic dysfunction. In the EASL-CLIF consensus, patients with acute decompensated cirrhosis were classified in three grades according to renal impairment and/or others extra-hepatic organ failures.

A better definition of ACLF could be obtained using new biomarkers. Nevertheless, it is now widely accepted that the search for a single biomarker that can be used in routine clinical practice to diagnosis patients with ACLF is probably unrealistic.

Metabolomics, which is the study of metabolic changes in an integrated biological system using multiparametric analyses, may help identify biomarkers that characterize the metabolic profiles of a disease, and/or evaluate metabolic modifications after treatment has been initiated [5]. Metabolomics, using proton nuclear magnetic resonance (^1^H-NMR) spectroscopy, when applied to liver disease, has shown a close relationship between metabolic abnormalities and the severity of the disease in sera and tissues [6], [7], [8].

Jimenez *et al.* have shown that using ^1^H-NMR to assess the metabolomics of sera from patients with cirrhosis could discriminate between patients with minimal hepatic encephalopathy and those with no encephalopathy [9]. Other studies show that metabolomics can fingerprint the differences between compensated and decompensated cirrhosis, and between cirrhosis caused by alcohol or viruses [10], [11]. We have previously shown that a metabolomic approach was a powerful tool in assessing the severity of chronic liver failure in alcohol-induced cirrhosis within a cohort of patients without ACLF. In our previous study, the severity of chronic liver failure was evaluated using the MELD score, and correlated well with impairment of lipid, glucose, and amino acid metabolism [6].

In ICU investigations of liver disease, some studies have evaluated the different metabolic profiles after transplantation and have compared non-survivor to survivor or graft-failure cases: they have shown improvement in patients’ liver status after transplantation [12], [13]. Another study that assessed sera using ^1^H-NMR showed the potential of the metabolomic profile to evaluate patients with fulminant hepatic failure and to predict an unfavorable outcome [14]. However, none of these studies have focused on the changes in metabolism that occur in patients with cirrhosis and ACLF.

We hypothesize that patients with cirrhosis and hospitalized in ICUs have acute changes to their metabolism that can be identified using ^1^H-NMR to evaluate their sera. These metabolic changes could then identify patients with ACLF, independently of trigger events. Thus, the purpose of our study was to assess whether the metabolomic profiles of sera, obtained by ^1^H-NMR spectroscopy, were modified in patients with alcohol-induced cirrhosis and hospitalized in an ICU for an acute event known to cause ACLF, when compared to patients with compensated or decompensated, but stable, cirrhosis. Another aim was to identify the impaired common metabolic pathways. Consequently, the decrease or the increase of the different metabolic pathways will be discussed.

## Materials and Methods

### Patients and Collection of Sera Samples

Between August 2010 and December 2010, all consecutive patients with cirrhosis and referred to our Liver Center were screened for the study. Unstable cirrhotic patients with high risk of liver and/or other organ failure secondary to a severe acute event, mainly gastrointestinal bleeding (GIB) or sepsis, hospitalized and managed in ICU were included in the ACLF group. Asian or EASL-CLIF consortium definitions were not used to include patient in ACLF group. Compensated and decompensated patients who had stable cirrhosis, and who had been referred to the hepatology unit, within the same period, for either therapeutic management (paracenthesis) or a screening procedure were included in the CLF group.

Cirrhosis was defined by a liver biopsy or the presence of at least two compatible diagnostic factors from the following: radiology (ultrasound, computed tomography, or magnetic-resonance imaging), clinical examinations (at least two indicators: hepatomegaly, splenomegaly, encephalopathy, at least two arterial spiders, collateral venous circulation, or jaundice), and/or biological factors (increased bilirubinemia, a prolonged International Normalized Ratio, or thrombocytopenia).

Only patients with alcoholic cirrhosis were considered. Exclusion criteria were infection with the human immunodeficiency virus, hepatitis B or C viruses, evidence of hepatocarcinoma (as judged by ultrasonographic findings and serum α-fetoprotein concentration >50 ng/mL), and a past history of acute decompensation during the previous 6 months.

The date of inclusion into the study was the date when blood was collected. Blood samples were drawn under fasting conditions for the CLF group and immediately at admission for the ALCF group. Serum was separated and stored at –80°C until further analyses. Gender, age, the presence of ascites or hepatic encephalopathy, serum bilirubin, albumin and prothrombin levels, the International Normalized Ratio, serum alanine-aminotransferase (ALT) activity, serum aspartate-aminotransferase (AST) activity, creatininemia, and platelet counts, were recorded at inclusion.

For patients with ALCF, diagnosis was made as they entered the ICU ward. The main diagnoses were sepsis, GIB, acute renal failure, or hepatic encephalopathy. Gastrointestinal bleeding was defined as any hematemesis or melena with transfusion of two or more units of blood. Sepsis was defined as proposed in the International Survey Sepsis Campaign [15]. Blood lactate, hemodynamic parameters, and the use of catecholamine and mechanical ventilation were recorded. To evaluate the severity of the disease, the Sequential Organ Failure Assessment (SOFA) score was calculated during the first day of admission [16]. For all patients, the severity of chronic liver failure was calculated using both the Child–Pugh–Turcott and MELD scores [17], [18]. For the ACLF group, the more recent Child–Pugh–Turcott score before hospitalization in the ICU was taken into account.

### Ethics Statement

The Institutional Review Board of Jean Verdier University Hospital approved the protocol and the French Research Delegation Office accepted the creation of a dedicated bio-collection for the patients included in the protocol. The CNIL (National Informatics and Liberty Commission) also approved the creation of both bio-collection and database. Depending of the site of hospitalization (ward or ICU) and the mental status of the patient, the written consent was obtained directly from the patient at inclusion or from the Person of Confidence designed by the patient (pre-emptively) or by the family relatives. In case, the patient survived, secondary written consent from the patient was systematically seek.

### ^1^H-NMR Spectroscopy

For NMR analysis, samples were thawed at room temperature. A volume of 0.6 mL of serum was placed into a 5-mm-diameter tube together with 0.1 mL of D_2_O, which contained a known amount of fumaric acid. The proton spectra were acquired at 500 MHz on a Varian Unity Inova® spectrometer at 25°C. The signal was acquired after a 90° pulse on 32-K data points for a spectral window of 5000 Hz. The relaxation delay was 4 s. The water signal was suppressed by a pre-saturation sequence using low-power irradiation (0.03 W for 2 s) on the water-signal frequency during the relaxation delay. The Carr-Purcell-Meiboom-Gill (CPMG) sequence is frequently used by others [9] to suppress the broad protein signal according to its short T2 relaxation time. Because lipids content in the sera of patients with cirrhosis [6] are an important discriminant variable, a single pulse sequence was preferred to preserve the complete lipid profile of the spectra. The resulting free induction decays obtained with 128 transients were processed by Mestrec® software.

A Fourier transformation was applied with an exponential window function to produce a 1-Hz broadening line. The spectra were phased and a spline baseline correction was applied with three points at 0.5, 4.6, and 9 ppm. The chemical shifts were referenced using the fumaric-acid signal (6.53 ppm). The spectral region between 0–9 ppm was divided into 9000 spectral regions of 0.001-ppm width, called buckets, using a personal program with R®. Water, urea (signal damaged by water-saturation transfer), and fumaric-acid regions were excluded (1810 variables excluded).

At admission, ICU patients were not fasted whereas patients from the CLF group were. Moreover, some ICU patients were perfused with hydroxyl-ethyl starch, a volume expender, before admission to the ICU. Glucose residues of hydroxyl-ethyl starch produce an intense and broad resonance between 3.35 and 4.06 ppm. For this reason, the region between 3.35 and 4.06 ppm, corresponding to the glucose signal, was excluded from all spectra of the CLF and ACLF groups. Each bucket integral was integrated and scaled to the total summed bucket integrals for each spectrum.

### Demographic and Biological Analyses of Data

Qualitative variables were compared using Fischer’s exact, chi-squared test or the chi-squared trend test with 1 degree of freedom, whereas quantitative variables were compared using the non-parametric Wilcoxon’s test. All reported *p* values are two-tailed. Associations were considered to be statistically significant at two-tailed α-values of 0.05.

### Multivariate Analyses

A principal component analysis (PCA) was first performed to detect any group separation based on NMR-signal variability. This method also enabled detection and exclusion of any outliers, defined as observations located outside the 95% confidence region of the model. Orthogonal projection to latent-structure (OPLS) analysis was run to identify differences between patients in the CLF and ACLF groups. Compared to the classical projection of latent-structure analysis, this method allowed improved interpretation of the spectroscopic variations between the groups, by removing information that had no impact on the differences. PCA and OPLS analyses were performed using Simca-P12 (Umetrics, Umea) and in-house Matlab® (Mathworks, Natick, MA) code based on the Trygg and Wold method [19].

The goodness-of-fit parameters for the OPLS model, *R* ^2^Yand *Q* ^2^Y, were calculated. *R* ^2^Y represents the explained variance of the Y matrix. *Q* ^2^Y estimates the predictability of the model. *R* ^2^Y = 1 indicates perfect description of the data by the model, whereas *Q* ^2^Y = 1 indicates perfect predictability. For internal validation of the OPLS models, a permutation test (999 permutations) was performed. This evaluated whether the OPLS models, built with the groups, was significantly better than any other OPLS model obtained by randomly permuting the original group attributes.

A score plot illustrated the results. Each point in the score-plot represents the projection of an NMR spectrum (and thus a patient’s sample) on the predictive (horizontal axis) and the first orthogonal component of the model (vertical axis). The loading plot represents the covariance between the Y-response matrix and the signal intensity of the various spectral domains. Colors were also used in the loading plot depending of the *p-*value associated with the correlation between the corresponding bucket intensity and Y variable.

The null hypothesis associated with the *p*-values used in this study was that there was no correlation between X (intensity of the buckets) and Y (CLF or ALCF group) variables. To minimize false-positive rates in multiple comparisons of the 4239 spectral domains, we used the conservative Bonferroni correction, which discards any low significant variables. Thus, for an error rate of 0.01, a metabolite variation in the loading plot was considered significant if its *p-*value was less 2.4×10^–6^.

### Identification of Metabolites

On the loading plot, positive signals corresponded to the metabolites that had increased concentrations in the sera from patients in the ALCF group. Conversely, a negative signal corresponded to metabolites that had an increased concentration in the sera of patients in the CLF group.

The buckets were designated according to their central chemical-shift values. Their most probable assignment to a specific metabolite was given according to the spectral assignment and its detailed appearance (multiplet, high-resolution peaks, and broad signals), as previously described in the literature [20], [21], and according to our previous signal assignments [6], [22]. To help identify the metabolites, statistical analysis of total-correlation spectroscopy was performed (data not shown) [23].

## Results

### Patients’ Characteristics and PCA Analysis

**Figure 1:**
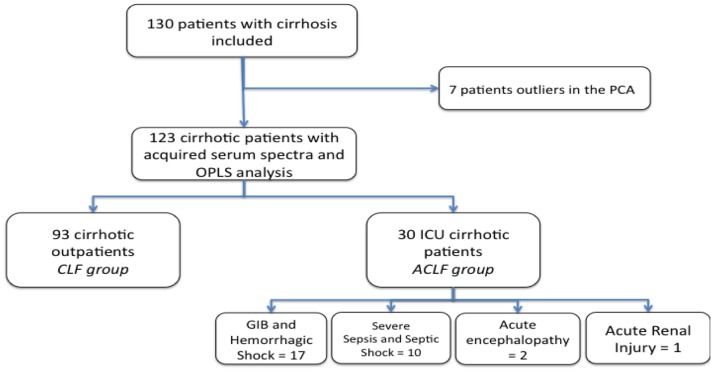
Flow chart of the patients.

A total of 130 patients fulfilled the inclusion criteria: there were 35 patients in the ACLF group and 95 patients in the CLF group. A flow chart of the patients’ characteristics is presented in Figure 1. The data obtained by bucketing the spectra of 130 sera were first analyzed by PCA (not shown). From this analysis, seven outliers were identified: two in the CLF group and five in the ACLF group. The two outliers in the CLF group exhibited high levels of ethanol: 1.11 and 3.66 ppm. Of the five outliers in the ACLF group, three concerned hemorrhagic shock previously transfused. All these outliers were excluded from the OPLS analysis.

Demographic, clinical, and biological features of all patients included in the study are displayed in Table 1, and specific characteristics of the ICU patients included in the OPLS analysis are displayed in Table 2.

**Table 1:** Baseline characteristics of the overall population.

Factor	CLF group(*n* = 95)	ACLF group(*n* = 35)
**Age (years)** a	58.1±1.0	58.9±1.5
**Male gender** b	85 (84%)	27 (87%)
**ALT (UI/L)** a	54.6±6.0	142.3±69.5*
**AST (UI/L)** a	94.5±14.5	532.5±275.5*
**Albumin (g/L)** a	37.1±0.7	31.4±1.2*
**Prothrombin level (% control)** a	65.7±2.2	34.7±2.8*
**Bilirubin (µmol/L)** a	45.3±7.6	75.2±14.4
**Child–Pugh score** a	7.0±0.2	9.6±0.3*
**MELD score** a	13.4±0.6	25.6±1.8*
**Creatinine (µmol/L)** a	85.3±3.5	158.6±18.8*
**Blood glucose (mmol/L)**	6.6±0.2	8.3±0.8*
**White blood-cell count (G/L)** a	6.1±0.2	12.6±1.6*
**Paletted count (G/L)** a	137.7±7.5	101.4±12.9*
**Hemoglobinemia (g/L)**	12.6±0.2	8.6±0.4*

Note. All biological and clinical parameters were recorded at inclusion.

a Mean ± SEM.

b Number (percentage) of patients.

*p<0.05 between ACLF and CLF groups.

**Table 2:** Characteristics of patients with ACLF included in the OPLS analysis.

Initially diagnosed in the ICU	30
GIB	12
Hemorrhagic shock	5
Severe sepsis	4
Septic shock	6
Acute kidney failure	1
Hepatic encephalopathy	2
**Previous CPT**	7.6±2.1
**Admission CPT**	9.7±2.0*
**Mechanical ventilation (n)**	16/30
**Catecholamines (n)**	7/30
**SOFA**	7.7±3.6
**Encephalopathy (0/I/II/III/IV)**	3/11/10/1/6
**Lactate (mM)**	5.7±6.3
**Death (n)**	6/30

*p<0.01 between previous and admission CPTs.

Notes: SOFA: Sequential Organ Failure Assessment; CPT: Child–Pugh–Turcott score; GIB: gastrointestinal bleeding.

### OPLS Model and Their Validation

**Figure 2:**
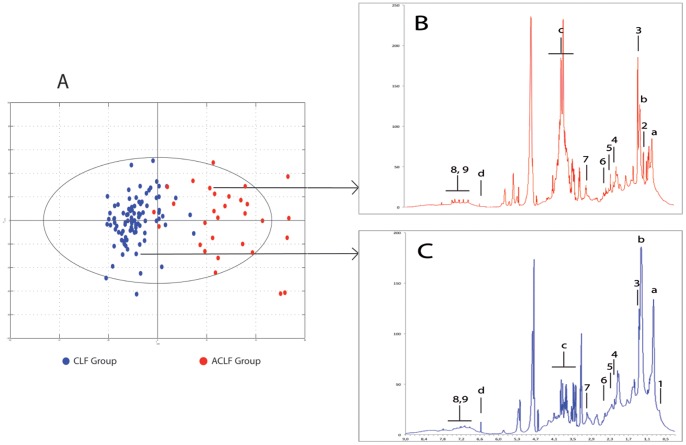
OPLS score plot and spectra of the CLF and ACLF patients. A: Score plot separating CLF (blue) and ACLF (red) patients. B and C: Representative spectra showing the metabolic differences between patients with CLF or ACLF. Legends: a: -CH_3_ of fatty acid; b; -CH_2_ of fatty acid; c: glucose (C) and hydroxy-ethyl starch (B); d: fumaric acid; 1: cholesterol; 2: hydroxybutyrate; 3: lactate; 4: acetoacetate; 5: pyruvate; 6: glutamine; 7: creatinine; 8 & 9: aromatic amino acids (phenylalanine and tyrosine).

Spectra of the sera from the CLF and ACLF groups were identified using the OPLS model, as shown in the score plot in Figure 2A. Examples of typical spectra from each group are shown in Figure 2B and C. The model was built with one predictive and two Y-orthogonal components, and exhibited a good explained variance (*R* ^2^Y) of 0.73 and a predictability (Q^2^ *Y*) of 0.63.

On the score plot, the spectra from the CLF and ACLF groups were well separated along the horizontal axis. Nevertheless, four patients from the ACLF group were misclassified and had similar metabolomic profiles to patients from the CLF group: two patients with ACLF had been hospitalized in the ICU for gastrointestinal bleeding, which was not related to portal hypertension and was not severe, one had acute kidney injury related to acute cardiac insufficiency, and one had an acute episode of seizure related to alcohol withdrawal. All these patients were alive after hospitalization in the ICU, and none had major impairment of liver function during their stay in the ICU. Two patients from the CLF group were misclassified on the score plot in the ACLF group: one had liver and kidney failure and died at 1 month after inclusion, and the other was initially classified as Child A at admission although the final diagnosis was non-severe acute alcoholic hepatitis.

The model was internally validated, as all *Q* ^2^Y and *R* ^2^Y values obtained with the permuted Y were smaller than those from the model. Intercept values for *Q* ^2^Y and *R* ^2^Y, obtained from the permutation plot (not shown), were, respectively, −0.22 and 0.19.

To evaluate that the difference of the group size did not influence our results, we have built 500 different models. Those OPLS models were built with 30 patients of CLF group randomly selected and 30 ACLF cases. The distribution of Q2Y values obtained described the results. The median of this Q2Y distribution was 0.64 and the smaller Q2Y value was 0.49. For each NMR variable, distribution of the regression coefficient was also estimated. The variables considered as significant in the original model were also significant in the 500 resampled models.

### Discriminants Metabolites

**Figure 3:**
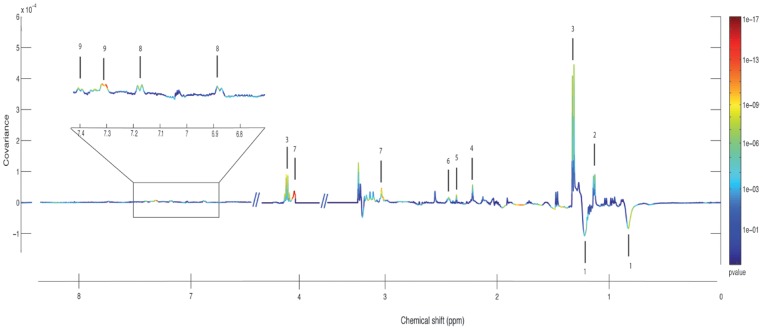
OPLS line plot showing the discriminant metabolites between patients with CLF or ACLF. Variations of metabolites are represented using a line plot between 0–9 ppm. Positive signals correspond to metabolites present at increased concentrations in the ACLF group. Conversely, negative signals correspond to metabolites present at increased concentrations in CLF group. The buckets are labeled according to metabolite assignment, as presented in Table 3.

The metabolites that differed between the groups could be identified within the sera spectra according to their loading plots (Figure 3). Table 3 presents the nine metabolites that were significantly correlated with the model and their variations according to group.

**Table 3:** Discriminant metabolites observed by ^1^H-NMR spectroscopy.

No. of metabolite	Name	Chemical shift (ppm) and multiplicity$	*r* #	*p* *	Group
**1**	Lipids (mainly HDL)	0.8^b^; 1.2^b^	−0.46	4,8×10^−8^	CLF
**2**	Hydroxybutyrate	1.13^d^	+0.38	1.7×10^−6^	ACLF
**3**	Lactate	1.30^d^; 4.13^q^	+0.55	2.6×10^−11^	ACLF
**4**	Acetoacetate	2.11^s^	+0.43	3.1×10^−7^	ACLF
**5**	Pyruvate	2.36^s^	+0.54	4.9×10^−11^	ACLF
**6**	Glutamine, glutamate	2.42^m^	+0.48	9.6×10^−9^	ACLF
**7**	Creatinine	3.03^s^ 4.04^s^	+0.56	1.1×10^−12^	ACLF
**8**	Tyrosine	6.88^m^;7.18^d^	+0.45	9.0×10^−8^	ACLF
**9**	Phenylalanine	7.31^m^; 7.40^m^	+0.63	3.3×10^−15^	ACLF

The principal discriminant metabolites are ordered according to the loading plots of patients with CLF and ACLF. The number of the metabolite corresponds to the number in the line plot in Figure 2. The chemical shift and multiplicity correspond to those found in the ^1^H-NMR spectra of the patients’ sera.

$ ppm: parts per million. s, singlet; b, broad; d, doublet; dd, doublet of doublet; t, triplet, m multiplet; q, quadriplet.

# A positive correlation (*r*) indicates an increased concentration in the ACLF group and a negative correlation indicates a decreased concentration in the CLF group.

*A *p*-value <2.4×10^−6^ is significant.

In the ^1^H-NMR spectra of sera, the lipids were detected as broad resonances of fatty-acid methyl and methylene moieties at 0.8 and 1.24 ppm, respectively, and the N-trimethyl moiety of choline was included in phospholipids at 3.2 ppm. The line shape of the methyl and methylene fatty-acid resonances, at 0.8 and 1.24 ppm, depended on the size of the lipoprotein particles. It has been widely reported that high-density lipid (HDL) particles produce resonances with lower chemical shifts than low-density lipoprotein (LDL and VLDL) particles. Interestingly, lipoproteins with higher densities were significantly elevated in the CLF group. For VLDL, corresponding to the highest chemical shifts in methyl and methylene resonances, no differences were found.

Lactate was an important metabolite that discriminated between the ACLF and CLF groups. Patients with ACLF had significantly higher lactate signals than patients in the CLF group. Pyruvate and ketone bodies (mainly acetoacetate) were significantly higher in the ACLF group than in CLF group.

Several amino acids could be identified in the spectra. Glutamine and glutamate were significantly higher in the ACLF group than in the CLF group. Aromatic amino acids, such tyrosine and phenylalanine, were significantly higher in the ACLF group compared to the CLF group. Creatinine was also higher in the spectra of patients in the ACLF group compared to the CLF group.

## Discussion

This preliminary study was conducted in a cohort of cirrhotic patients hospitalized in ICU for acute event and cirrhotic outpatients with compensated or decompensated but stable liver function. This investigation clearly shows a difference in the metabolomic profiles of the two studied populations. The comparison of these two populations of cirrhotic patients was preferred than comparison of ACLF cirrhotic patient and healthy volunteers. In the last case, metabolomics difference should be only due to chronic liver failure and not to acute impairment of the liver function.

During the evolution of chronic liver failure, several changes in liver metabolism were detected. In our previous study, stable patients with alcoholic cirrhosis and various degrees of liver injury (evaluated by the MELD score) were included. In severe chronic liver failure, metabolism of lipids, glucose, and ketone bodies were significantly discriminants [6]. Interestingly, in the present study, the metabolomic profile of ACLF patients was not the same metabolomic profile than determine in CLF group and in our previous study (6). In the ACLF group, lipid metabolism, lactate, amino-acid metabolism, and urea metabolism were affected by impaired liver function. This point is important as it differentiated the metabolomic fingerprints of chronic liver failure in acute-on-chronic liver failure.

In the CLF group, HDL signal were higher than in the ACLF group, but there was no difference with regards to VLDL and LDL. This is probably because ACLF patients were not fasted whereas those in the CLF group were. Thus, this may have caused the lipid signals to be higher in ACLF sera. Despite this discrepancy, the resonances corresponding to lipids were not increased in ACLF patients. Moreover, the HDL-lipid region was lower in ACLF sera compared to that of fasting patients in the CLF group. In several studies, higher HDL levels seem to reflect good hepatic function. The liver plays a key role in lipid metabolism, and low HDL levels seem to be caused by the severity of the chronic liver disease rather than indicating an acute episode of chronic impairment. A rise of serum-lactate concentration is common in cases of acute liver failure and has been proposed as a prognostic marker [24]. The production of lactate is also impaired in chronic liver failure accompanied by a decrease in hepatic pyruvate dehydrogenase activity [25]. Moreover, lactate is a major biomarker for tissue hypoxia and necrosis. Hepatic necrosis and inflammation, detected by histological examination of a liver biopsy, correlate well with the severity of ACLF and a poor outcome [26]. In our previous study, lactate was a key-indicator metabolite in patients with cirrhosis and the highest Model for End-Stage Liver-Disease scores. In patients with ACLF, increased lactate concentration may be related to the superimposed impairment of hepatic lactate catabolism and increased production of lactate caused by hypoxia and liver necrosis. Ketone bodies (including hydroxybutyrate and acetoacetate) were also increased in ACLF sera. These metabolites mainly originate from the metabolism of fatty acids via the beta-oxidation pathway, which produces acetyl moieties that enter the TCA cycle. Taken together, a decrease in HDL and an increase in lactate and ketone bodies have led to the hypothesis that aerobic glycolysis is modulated towards anaerobic and lipid metabolisms.

The perturbation of the aromatic acids metabolism (phenylalanine and tyrosine metabolism), has been found in different etiologies of liver injury, such as traumatic injury, and seems to be early markers [27]. In patients with chronic liver disease and who have had an acute event, such as gastrointestinal bleeding, infection, acute alcoholic hepatitis, or encephalopathy, the aromatic acids are increased in relation to inflammation and hepatic necrosis [28]. Systemic inflammation occurs in patients with cirrhosis who have a superimposed liver insult, and is significantly related to bleeding, sepsis, and long-term outcomes [29]. Inflammation plays a key role in worsening the patient’s health, and elevated levels of multiple pro-inflammatory cytokines have been described in ACLF [30].

The liver is the major organ for urea metabolism. Impaired urea metabolism leads to decreased ammonia detoxification and is responsible for the increase levels of glutamine and glutamate. This increase occurs in different situations in patients with chronic liver diseases: gastrointestinal bleeding, infection [28], hepatic encephalopathy, or end-stage liver disease [6].

Then, our metabolomic approach identifies several indicative factors within one experiment, whereas these factors have been only previously described in a range of situations in patients with cirrhosis. The fact that lactate and creatinine, for example, represents established biomarkers in the management of decompensated cirrhosis is not novel at all. The improvement reach by the metabolomics approach could be the simultaneous evaluation of the metabolites of interest in a profile directly correlated to the clinical status.

Two patients within our CLF group were misclassified in the score plot and had similar metabolomic profile than patients of ACLF group. Both patients had impaired liver function: this was caused by acute alcoholic hepatitis for one, and cardiac failure for the other. Metabolomic fingerprints seemed able to reflect changes in liver function before standard biological laboratory tests could. In addition, four patients from the ACLF group were misclassified as being in the CLF group. They had minor gastrointestinal bleeding, acute kidney injury, encephalopathy that was not related to liver disease, and none or minor liver injury. Metabolomic profiles could excluded acute liver damage better than standard biological laboratory test.

The main limitation encountered in a translational metabolomic approach to liver disease is methodological bias when selecting patients, which limits the confidence that can be drawn from conclusions. We attempted to avoid these pitfalls by constructing well-defined subgroups of patients according to their stage of underlying liver disease, with a control group composed of compensated or uncompensated (CLF group), but stable, cirrhosis, and a group of patients with cirrhosis who had an acute event that was responsible for the superimposed liver impairment (ACLF group). We also focused specifically on the etiology of alcoholism to avoid any potential specific influence of viral or non-alcoholic-related liver disease on the metabolomic profiles of the sera, as has been previously reported [10].

In this setting, the present data enabled us to form several hypotheses and conclusions. First, the metabolomic fingerprints reflect major changes to liver function, such as energy metabolism, amino-acid metabolism, and urea metabolism. Secondly, in cases of acute impairment, the changes correlated with the severity of liver failure (as shown in our previous study) and they were increased. Other modulations were not found in chronic liver failure and increase in case of acute dysfunction of the liver such as the aromatic acids. Third, increase creatinine in the ACLF group reflects extra-liver failure with renal injury comparatively of cirrhotic patients of the CLF group. Fourth, the metabolomic changes were related to inflammation and necrosis. Systemic inflammation and necrosis are known to play a major role in the pathophysiology of acute-on-chronic liver failure and the failure of other organs. In addition, our metabolomic profiles improved the classification of patients with or without acute-on-chronic liver failure, as was shown with the initially misclassified patients.

A metabolomic approach assesses the liver markers and other organ impairments in serum samples using a unique experiment: this profiling provides complete and multivariate information on the metabolomic changes related to ACLF and can stratify the cases. The use of this technique as a diagnostic and prognostic tool for patients with ACLF needs to be further investigated. Finally, further confirmation of our metabolomic findings needs to be tested in larger prospective cohorts of ICU patients being followed up for cirrhosis in order to better define clear association between metabolomic profiles and diagnosis or prognosis. Such an approach will improve our understanding of the involvement of the biological pathways in ACLF.

## References

[1] Olson JC, Wendon JA, Kramer DJ, Arroyo V, Jalan R, et al (2011) Intensive care of the patient with cirrhosis. Hepatology 54: 1864–1872. 21898477 10.1002/hep.24622

[2] Jalan R, Williams R (2002) Acute-on-chronic liver failure: pathophysiological basis of therapeutic options. Blood purification 20: 252–261. 11867872 10.1159/000047017

[3] Moreau R, Jalan R, Gines P, Pavesi M, Angeli P, et al.. (2013) Acute-on-chronic liver failure is a distinct syndrome that develops in patients with acute decompensation of cirrhosis. Gastroenterology 144: 1426–1437, 1437 e1421–1429. 10.1053/j.gastro.2013.02.042 23474284

[4] Sarin SK, Kumar A, Almeida JA, Chawla YK, Fan ST, et al (2009) Acute-on-chronic liver failure: consensus recommendations of the Asian Pacific Association for the study of the liver (APASL). Hepatology international 3: 269–282. 19669378 10.1007/s12072-008-9106-x PMC2712314

[5] Dunn WB, Broadhurst DI, Atherton HJ, Goodacre R, Griffin JL (2011) Systems level studies of mammalian metabolomes: the roles of mass spectrometry and nuclear magnetic resonance spectroscopy. Chemical Society reviews 40: 387–426. 20717559 10.1039/b906712b

[6] Amathieu R, Nahon P, Triba M, Bouchemal N, Trinchet JC, et al (2011) Metabolomic approach by 1H NMR spectroscopy of serum for the assessment of chronic liver failure in patients with cirrhosis. Journal of proteome research 10: 3239–3245. 21568267 10.1021/pr200265z

[7] Martinez-Granados B, Morales JM, Rodrigo JM, Del Olmo J, Serra MA, et al (2011) Metabolic profile of chronic liver disease by NMR spectroscopy of human biopsies. International journal of molecular medicine 27: 111–117. 21072494 10.3892/ijmm.2010.563

[8] Yu K, Sheng G, Sheng J, Chen Y, Xu W, et al (2007) A metabonomic investigation on the biochemical perturbation in liver failure patients caused by hepatitis B virus. Journal of proteome research 6: 2413–2419. 17539670 10.1021/pr060591d

[9] Jimenez B, Montoliu C, MacIntyre DA, Serra MA, Wassel A, et al (2010) Serum metabolic signature of minimal hepatic encephalopathy by (1)H-nuclear magnetic resonance. Journal of proteome research 9: 5180–5187. 20690770 10.1021/pr100486e

[10] Qi S, Tu Z, Ouyang X, Wang L, Peng W, et al (2012) Comparison of the metabolic profiling of hepatitis B virus-infected cirrhosis and alcoholic cirrhosis patients by using (1) H NMR-based metabonomics. Hepatology research : the official journal of the Japan Society of Hepatology 42: 677–685. 22404306 10.1111/j.1872-034X.2011.00964.x

[11] Qi SW, Tu ZG, Peng WJ, Wang LX, Ou-Yang X, et al (2012) (1)H NMR-based serum metabolic profiling in compensated and decompensated cirrhosis. World journal of gastroenterology : WJG 18: 285–290. 22294833 10.3748/wjg.v18.i3.285 PMC3261547

[12] Tripathi P, Bala L, Saxena R, Yachha SK, Roy R, et al (2009) 1H NMR spectroscopic study of blood serum for the assessment of liver function in liver transplant patients. Journal of gastrointestinal and liver diseases : JGLD 18: 329–336. 19795028

[13] Serkova NJ, Zhang Y, Coatney JL, Hunter L, Wachs ME, et al (2007) Early detection of graft failure using the blood metabolic profile of a liver recipient. Transplantation 83: 517–521. 17318087 10.1097/01.tp.0000251649.01148.f8 PMC2709529

[14] Saxena V, Gupta A, Nagana Gowda GA, Saxena R, Yachha SK, et al (2006) 1H NMR spectroscopy for the prediction of therapeutic outcome in patients with fulminant hepatic failure. NMR in biomedicine 19: 521–526. 16598697 10.1002/nbm.1034

[15] Dellinger RP, Levy MM, Carlet JM, Bion J, Parker MM, et al (2008) Surviving Sepsis Campaign: international guidelines for management of severe sepsis and septic shock: 2008. Critical care medicine 36: 296–327. 18158437 10.1097/01.CCM.0000298158.12101.41

[16] Vincent JL, de Mendonca A, Cantraine F, Moreno R, Takala J, et al (1998) Use of the SOFA score to assess the incidence of organ dysfunction/failure in intensive care units: results of a multicenter, prospective study. Working group on “sepsis-related problems” of the European Society of Intensive Care Medicine. Critical care medicine 26: 1793–1800. 9824069 10.1097/00003246-199811000-00016

[17] Pugh RN, Murray-Lyon IM, Dawson JL, Pietroni MC, Williams R (1973) Transection of the oesophagus for bleeding oesophageal varices. The British journal of surgery 60: 646–649. 4541913 10.1002/bjs.1800600817

[18] Kamath PS, Wiesner RH, Malinchoc M, Kremers W, Therneau TM, et al (2001) A model to predict survival in patients with end-stage liver disease. Hepatology 33: 464–470. 11172350 10.1053/jhep.2001.22172

[19] Trygg J, Wold S (2002) Othogonal projections to latent structure (OPLS). J Chemometrics 16: 116–128.

[20] Nicholson JK, Foxall PJ, Spraul M, Farrant RD, Lindon JC (1995) 750 MHz 1H and 1H-13C NMR spectroscopy of human blood plasma. Anal Chem 67: 793–811. 7762816 10.1021/ac00101a004

[21] Wevers RA, Engelke U, Heerschap A (1994) High-resolution 1H-NMR spectroscopy of blood plasma for metabolic studies. Clin Chem 40: 1245–1250. 8013094

[22] Nahon P, Amathieu R, Triba MN, Bouchemal N, Nault JC, et al (2012) Identification of serum proton NMR metabolomic fingerprints associated with hepatocellular carcinoma in patients with alcoholic cirrhosis. Clinical cancer research : an official journal of the American Association for Cancer Research 18: 6714–6722. 23136190 10.1158/1078-0432.CCR-12-1099

[23] Cloarec O, Dumas ME, Craig A, Barton RH, Trygg J, et al (2005) Statistical total correlation spectroscopy: an exploratory approach for latent biomarker identification from metabolic 1H NMR data sets. Anal Chem 77: 1282–1289. 15732908 10.1021/ac048630x

[24] Bernal W, Donaldson N, Wyncoll D, Wendon J (2002) Blood lactate as an early predictor of outcome in paracetamol-induced acute liver failure: a cohort study. Lancet 359: 558–563. 11867109 10.1016/S0140-6736(02)07743-7

[25] Shangraw RE, Rabkin JM, Lopaschuk GD (1998) Hepatic pyruvate dehydrogenase activity in humans: effect of cirrhosis, transplantation, and dichloroacetate. The American journal of physiology 274: G569–577. 9530159 10.1152/ajpgi.1998.274.3.G569

[26] Rastogi A, Kumar A, Sakhuja P, Bihari C, Gondal R, et al (2011) Liver histology as predictor of outcome in patients with acute-on-chronic liver failure (ACLF). Virchows Archiv : an international journal of pathology 459: 121–127. 21744153 10.1007/s00428-011-1115-9

[27] Ranjan P, Gupta A, Kumar S, Gowda GA, Ranjan A, et al (2006) Detection of new amino acid markers of liver trauma by proton nuclear magnetic resonance spectroscopy. Liver international : official journal of the International Association for the Study of the Liver 26: 703–707. 16842327 10.1111/j.1478-3231.2006.01283.x

[28] Rosen HM, Yoshimura N, Hodgman JM, Fischer JE (1977) Plasma amino acid patterns in hepatic encephalopathy of differing etiology. Gastroenterology 72: 483–487. 832796

[29] Cazzaniga M, Dionigi E, Gobbo G, Fioretti A, Monti V, et al (2009) The systemic inflammatory response syndrome in cirrhotic patients: relationship with their in-hospital outcome. Journal of hepatology 51: 475–482. 19560225 10.1016/j.jhep.2009.04.017

[30] Sen S, Davies NA, Mookerjee RP, Cheshire LM, Hodges SJ, et al (2004) Pathophysiological effects of albumin dialysis in acute-on-chronic liver failure: a randomized controlled study. Liver transplantation : official publication of the American Association for the Study of Liver Diseases and the International Liver Transplantation Society 10: 1109–1119. 10.1002/lt.20236 15350001

